# Relationship between dietary selenium intake and serum thyroid function measures in U.S. adults: Data from NHANES 2007–2012

**DOI:** 10.3389/fnut.2022.1002489

**Published:** 2022-10-10

**Authors:** Fang Liu, Kai Wang, Jiaqi Nie, Qianqian Feng, Xiaosong Li, Yichi Yang, Ming-Gang Deng, Huanhuan Zhou, Suqing Wang

**Affiliations:** ^1^School of Public Health, Wuhan University, Wuhan, China; ^2^Department of Public Health, Wuhan Fourth Hospital, Wuhan, China; ^3^Hubei Key Laboratory of Food Nutrition and Safety, Department of Nutrition and Food Hygiene, Tongji Medical College, Huazhong University of Science and Technology, Wuhan, China; ^4^School of Nursing, Wuhan University, Wuhan, China; ^5^Center for Chronic Disease Rehabilitation, School of Nursing, Wuhan University, Wuhan, China

**Keywords:** selenium, thyroid hormones, NHANES, sex, iodine status

## Abstract

Although numerous studies have explored the relationship between selenium intake and thyroid diseases, few epidemiological studies have investigated the association between selenium intake and thyroid hormones. Therefore, we conducted this analysis to investigate the association between dietary selenium intake and thyroid hormones. Our sample included 5,575 adults (age ≥ 20) years from the National Health and Nutrition Examination Survey (NHANES) 2007–2012. Thyroid hormones, including total triiodothyronine (T3), total thyroxine (T4), free T3 (FT3), free T4 (FT4), and thyroid-stimulating hormone (TSH), were detected. Multivariable linear regression models showed that log10-transformed selenium intake (LogSe) was negatively correlated with TT4 (β = −0.383, 95% CI: −0.695, −0.070) and TT4/TT3 (β = −0.003, 95% CI: −0.006, −0.0004) in U.S. adults. Besides, additional stratified analyses by sex demonstrated that LogSe was negatively associated with TT4 (β = −0.007, 95% CI: −0.013, −0.001) and TT4/TT3 (β = −0.664, 95% CI: −1.182, −0.146) and positively associated with FT4/TT4 (β = 0.031, 95% CI: 0.004, 0.059) in male adults. Meanwhile, subgroup analysis by iodine status showed that LogSe was negatively associated with TT4 (β = −0.006, 95% CI: −0.011, −0.002), FT4/FT3 (β = −0.011, 95% CI: −0.023, −0.00002) and TT4/TT3 (β = −0.456, 95% CI: −0.886, −0.026) in iodine sufficiency but not in iodine deficiency adults. Our results demonstrated that the increased dietary selenium intake was negatively correlated with TT4 and TT4/TT3 in U.S. adults. Furthermore, the association between dietary selenium intake and thyroid hormones was more pronounced in males and iodine sufficiency adults.

## Introduction

Thyroid hormones, mainly including triiodothyronine (T3) and thyroxine (T4), are present in numerous tissues and play important roles in maintaining the normal development of the brain, heart, and other organs by controlling energy expenditure and thermogenesis, modulating lipid profiles, and maintaining the normal reproductive function, etc (1). The biosynthesis and secretion of thyroxine (T4) and triiodothyronine (T3) in the thyroid are regulated by thyroid-stimulating hormone (TSH). In turn, the synthesis and release of TSH from the pituitary could also be regulated by T4 and T3 *via* a negative feedback loop. T4 and T3 exist in free and binding forms in peripheral blood, and the former enters target cells to exert their biological functions. In addition, T4 can be converted to T3 under the action of type 1 and 2 iodothyronine deiodinases (DIO1, DIO2) (2). Studies have shown that both thyroid hormone deficiency and excess lead to serious diseases. Thyroid hormone deficiency is associated with hypertension, dyslipidemia, and coronary heart disease (CHD) (3, 4), while thyroid hormone excess is associated with atrial fibrillation (AF) and heart failure (HF) (5).

Selenium, an essential micronutrient, enters the human food chain through plants, seafood, and animal products (6). According to a recent study, the individual dietary selenium intake ranged from 7 to 4,990 μg/day due to varying selenium content in the soil in which crops were grown (7). Schwarz and Foltz demonstrated that a low concentration of selenium could help prevent hepatic necrosis as early as 1957, which was the first study to demonstrate the nutritional value of selenium (8). In recent years, some studies started to study the relationship between selenium and thyroid function because of selenium's anti-inflammatory and antioxidant roles (9). Given that increased inflammation is believed to play an important role in thyroid dysfunction, selenium as an anti-inflammatory substance mediated by selenoproteins could improve thyroid function, which might be propitious to regulate the synthesis and secretion of thyroid hormones (10). Besides that, in the process of thyroid hormone synthesis, the excessive production of H_2_O_2_ by thyroid follicular epithelial cells might damage the normal function of thyroid function (11), and selenium or antioxidant selenoproteins are acknowledged to scavenge H_2_O_2_ (12), which suggests selenium might be beneficial to ameliorating thyroid dysfunction induced by excessive H_2_O_2_ and ultimately influence the synthesis and secretion of thyroid hormones. Also, some previous studies found that selenium was the nucleophilic atom in the DIO1 active site, which further highlights the important role of selenium in thyroid hormones (13).

The current studies mainly explored the relationship between selenium intake and thyroid diseases. In a prospective randomized placebo-controlled clinical trial of 70 female patients with autoimmune thyroid disease who received 200 mcg sodium selenite or placebo daily for 3 months, adjuvant sodium selenite treatment reduced serum thyroid peroxidase antibodies levels by 36% (14). A cross-sectional study conducted in China found that low selenium status was associated with an increased risk of autoimmune thyroiditis, subclinical hypothyroidism, hypothyroidism, and enlarged thyroid (15). A longitudinal study conducted in Brazil also reported that dietary selenium intake was inversely associated with subclinical hypothyroidism (16). However, fewer studies have examined the association between selenium and thyroid hormones, and current findings are inconsistent. A cross-sectional study in coastal fishermen and inland subjects from Latvia found higher plasma selenium level was associated with lower TSH, but not T3 and T4 (17). In a randomized controlled trial among 491 Danes, Kristian et al. found that selenium supplementation could affect thyroid function by reducing serum TSH and FT4 concentrations (18). In contrast, two other studies, one in the UK, and the other in New Zealand, found no association between selenium intervention and thyroid hormone concentrations (19, 20).

Based on the above background, this study aimed to evaluate the cross-sectional association between dietary selenium intake and serum thyroid hormone with data from the National Health and Nutrition Examination Survey (NHANES).

## Materials and methods

### Study population

The National Health and Nutrition Examination Surveys (NHANES) was a nationwide and ongoing cross-sectional survey conducted among the non-institutionalized US population. To assemble a sample of participants who were representative of the civilian non-institutionalized U.S. population, a repeated 2-year cycle survey with a complex multistage probability sampling design was used. Detailed information about the survey design and methods has been described elsewhere (21). The NHANES protocol was approved by the National Center for Health Statistics (NCHS) Research Ethics Review Board.

**Figure 1:**
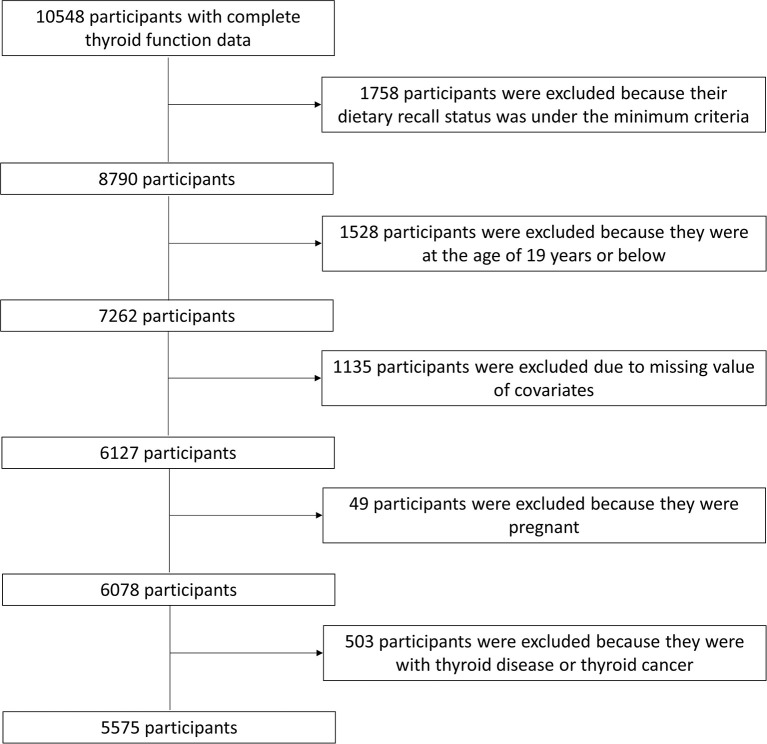
Flowchart of the study population.

Two thousand seven to two thousand eight, 2009–2010, and 2011–2012 NHANES cycles were selected, and a total of 10,548 participants with complete thyroid function data constituted the study sample. We excluded participants under the minimum criteria on dietary recall status (*n* = 1,758), at the age of 19 years or below (*n* = 1,528), with the missing value of covariates [education levels, household income, body mass index (BMI), urine iodine concentration (UIC), serum cotinine, and drinking (*n* = 1,135)]. Also, pregnant women (*n* = 49) and participants with thyroid disease or thyroid cancer (*n* = 503) were excluded. Finally, a total of 5,575 participants were included in the present study. These participants represented a weighted population of 73.5 million non-institutionalized US adults. The flowchart of sample selection was presented in Figure 1.

### Determination of serum thyroid hormones

During the physical examinations, whole blood specimens were collected into a red-top 15-mL vacutainer tube and then centrifuged, after which approximately 1 mL serum was collected for further biochemical examinations. Serum total T4 (TT4), total T3 (TT3), and free T3 (FT3) were determined using a competitive binding immunoenzymatic assay. Serum free T4 (FT4) was determined using a two-step enzyme immunoassay. Serum thyroid-stimulating hormone (TSH) was determined using a 3rd generation, two-site immunoenzymatic (“sandwich”) assay. Meanwhile, the ratios of FT4/FT3 and TT4/TT3 were calculated to reflect the metabolic level of peripheral T4, while the ratios of FT4/TT4 and FT3/TT3 were acquired to reveal the binding level of thyroid hormones with thyroid hormone-binding proteins (22).

### Dietary selenium intake assessment

In NHANES, dietary intake information was assessed by two reliable 24-h dietary recall interviews. The first dietary recall interview was conducted in the mobile examination center (MEC) and the second interview was conducted by telephone 3–10 days later. The intakes of dietary selenium during the 24-h period prior to the interview were calculated based on the University of Texas Food Intake Analysis System and U.S. Department of Agriculture (USDA) Survey Nutrients Database. Then, the mean value of selenium intake from the two 24-h dietary recall interviews was adopted as the final dietary selenium intake.

### Covariates

Potential covariates included age (20–39, 40–64, and ≥ 65) (23), sex (male, female), race (non-Hispanic White, non-Hispanic Black, Mexican American, and other races) (24), education level (less than a high school diploma, high school graduate/GED, some college/AA degree, and college graduate or more) (25), household income (family income to poverty (FPL) ≤ 1.3, 1, 3–3.5, and ≥ 3.5) (26), marital status (never married, married or living with a partner, and the other) (27), BMI (≤ 24.9, 25–29.9, and ≥ 30 kg/m^2^) (28), serum cotinine (< 1, 1–9.9, and ≥ 10 ng/mL) (1, 29), drinking (< 12, ≥ 12 times/year) (30), urine iodine concentration (≤ 100 and > 100 ug/L) (31), and fasting time (≤ 10 and > 10 h) (1).

Demographic data such as age, sex, race, education level, household income, and marital status were collected in an in-home interview. The body weight (kg) and height (m) were measured during mobile physical examination, and the BMI was calculated as BMI = weight/height^2^. Serum cotinine was measured using isotope dilution-high performance liquid chromatography/atmospheric pressure chemical ionization tandem mass spectrometry (ID HPLC-APCI MS/MS). Drinking status was ascertained *via* questionnaires. Urine iodine concentration was detected using inductively coupled plasma dynamic reaction cell mass spectroscopy (ICP-DRC-MS). In addition, fasting time was acquired by questionnaires before blood collection.

### Statistical analysis

In order to generate nationally representative estimates, SDMVPSU, and SDMVSTRA procedures were used to interpret NHANES's complex survey design, and WTMEC2YR was used to provide weight for all data.

The continuous variables were shown as means ± standard deviations, and the categorical variables were presented as counts (percentages). Initially, the Scott–Rao chi-square test was used to compare dietary selenium intake levels of different groups. Then, the one-way analysis of variance (ANOVA) was used to assess FT4, TT4, FT3, TT3, TSH, and ratios of thyroid hormones (FT4/FT3, TT4/TT3, FT4/TT4, and FT3/TT3) differences among different dietary selenium intake groups. Whereafter, two multivariable linear regression models were used to explore the association of dietary selenium intake with levels of FT4, TT4, FT3, TT3, TSH, FT4/FT3, TT4/TT3, FT4/TT4, and FT3/TT3. Due to right skewness, we log10-transformed dietary selenium intake to approximate normality assumptions. Model 1 (unadjusted) did not include any covariates. Model 2 was adjusted for sex, age, race, education level, household income, marital status, BMI, serum cotinine level, drinking, urine iodine concentration, and fasting time. Considering the effects of sex and urine iodine condition on thyroid function indices, we performed subgroup analyses to explore whether the association between dietary selenium intake and thyroid hormones was modified by sex and urine iodine condition. In addition, we carried out several restricted cubic spline (RCS) analyses to explore the non-linear dose-response relationship between dietary selenium intake and thyroid hormones in the whole and subgroup adults, and five knots were placed at the 5th, 25th, 50th, 75th, and 95th percentiles.

All statistical analyses were performed using STATA software (version 16.0), and R software (version 4.1.0, R Foundation for Statistical Computing). *P*-values and confidence intervals (CI) were reported two-sided without adjustment for multiple testing. The *p* < 0.05 was the significance criterion in the Scott–Rao chi-square test, ANOVA, and RCS analyses. Confidence intervals that do not contain 0 were considered to indicate statistical significance in multivariable linear regression models.

## Results

As shown in Table 1, participants were categorized according to their dietary selenium intake status. The chi-squared tests revealed dietary selenium intake was associated with sex, age, education level, marital status, household income, serum cotinine, drinking, and urine iodine concentration. The ANOVA analysis revealed that participants in the fourth quartile had significantly lower TT4, FT4/FT3, TT4/TT3, and higher FT3, TT3, FT4/TT4, and FT3/TT3 when compared to other groups.

**Table 1:** Characteristics of study participants.

**Characteristics**	**Dietary selenium intake (mcg/day)**				***p*-value**
	**Q1** **(0.000–77.950)**	**Q2 (77.950–105.550)**	**Q3 (105.550–140.100)**	**Q4 (140.100–523.450)**	
No. weighted %	1,560 (25.000)	1,411 (25.020)	1,362 (25.010)	1,242 (24.970)	
Sex					**< 0.001**
Male	498 (27.200)	644 (41.100)	856 (61.200)	1,007 (82.600)	
Female	1,062 (72.800)	767 (58.900)	506 (38.300)	235 (17.400)	
Age					**< 0.001**
20–39	416 (31.000)	450 (36.200)	469 (37.800)	516 (42.900)	
40–64	656 (47.300)	567 (43.400)	614 (48.400)	569 (49.400)	
≥65	488 (21.700)	394 (20.400)	279 (13.800)	157 (7.700)	
Race					0.063
Non-Hispanic white	731 (70.900)	700 (71.900)	674 (72.100)	598 (71.800)	
Non-Hispanic black	369 (12.800)	276 (9.900)	257 (9.500)	237 (9.500)	
Mexican American	234 (7.300)	211 (7.400)	208 (7.500)	196 (8.600)	
Other races	226 (8.900)	224 (10.800)	223 (10.800)	211 (10.100)	
Education level					**< 0.001**
< High school	532 (23.600)	368 (16.500)	317 (15.600)	280 (15.800)	
High school graduate/GED	381 (25.800)	339 (24.100)	316 (23.000)	269 (22.400)	
Some college/AA degree	391 (28.900)	405 (30.800)	397 (30.600)	357 (29.200)	
College graduate or more	256 (21.700)	299 (28.600)	332 (30.800)	336 (32.600)	
Marital status					**< 0.001**
Never married	249 (16.400)	231 (17.700)	221 (16.200)	251 (20.600)	
Married or living with a partner	881 (60.400)	833 (61.600)	885 (68.500)	812 (68.100)	
Others	430 (23.300)	347 (20.700)	256 (15.200)	179 (11.300)	
Household income					**< 0.001**
≤ 1.3 FPL	567 (25.000)	450 (22.200)	361 (16.100)	340 (18.200)	
1.3–3.5 FPL	610 (38.800)	533 (35.000)	515 (33.800)	447 (32.300)	
>3.5 FPL	383 (36.200)	428 (42.800)	486 (50.100)	455 (49.500)	
BMI status					0.224
≤ 24.9	428 (32.100)	404 (31.900)	345 (26.500)	363 (28.300)	
25–29.9	537 (33.500)	493 (34.300)	503 (37.200)	404 (35.300)	
>30	595 (34.400)	514 (33.900)	514 (36.300)	475 (36.400)	
Serum cotinine					**0.037**
< 1 ng/mL	1,106 (69.500)	1,006 (71.700)	980 (73.300)	816 (66.300)	
1–9.9 ng/mL	68 (4.000)	59 (3.700)	53 (4.000)	66 (5.800)	
≥10 ng/mL	386 (26.500)	346 (24.700)	329 (22.700)	360 (27.900)	
Drinking					**< 0.001**
< 12 times/year	570 (31.300)	405 (23.700)	285 (17.600)	224 (14.600)	
≥12 times/year	990 (68.700)	1,006 (76.300)	1,077 (82.400)	1,018 (85.400)	
Urine iodine concentration					**0.025**
>100 ug/L	1,038 (63.100)	962 (66.700)	917 (68.200)	876 (70.500)	
≤ 100 ug/L	522 (36.900)	449 (33.300)	445 (31.800)	366 (29.500)	
Fasting time					0.855
≤ 10 h	1,003 (64.800)	930 (66.800)	879 (66.000)	819 (65.600)	
>10 h	557 (35.200)	481 (33.200)	483 (34.000)	423 (34.400)	
FT4 (pmol/L)	10.150 ± 0.043	10.246 ± 0.052	10.100 ± 0.047	10.118 ± 0.051	0.138
TT4 (ug/dL)	7.922 ± 0.041	7.920 ± 0.040	7.650 ± 0.040	7.559 ± 0.043	**< 0.001**
FT3 (pg/mL)	3.137 ± 0.010	3.161 ± 0.010	3.186 ± 0.017	3.270 ± 0.010	**< 0.001**
TT3 (ng/dL)	113.606 ± 0.609	114.748 ± 0.630	112.279 ± 0.581	115.228 ± 0.626	**0.003**
TSH (mIU/I)	2.045 ± 0.053	1.945 ± 0.059	2.015 ± 0.052	1.870 ± 0.034	0.069
FT4/FT3	3.276 ± 0.017	3.273 ± 0.017	3.210 ± 0.017	3.125 ± 0.018	**< 0.001**
TT4/TT3	0.072 ± 0.000	0.071 ± 0.000	0.070 ± 0.000	0.067 ± 0.000	**< 0.001**
FT4/TT4	1.308 ± 0.006	1.318 ± 0.006	1.349 ± 0.007	1.365 ± 0.007	**< 0.001**
FT3/TT3	0.028 ± 0.000	0.028 ± 0.000	0.029 ± 0.000	0.029 ± 0.000	**< 0.001**

Q1, Quartile 1; Q2, Quartile 2; Q3, Quartile 3; Q4, Quartile 4. FPL, family income to poverty; BMI, body mass index; FT4, free thyroxine; TT4, total thyroxine; FT3, free triiodothyronine; TT3, total triiodothyronine; TSH, thyroid-stimulating hormone. The bold values indicate statistically significant differences.

In the unadjusted model (Table 2), dietary selenium intake, described as LogSe, negatively correlated with TT4 (β = −0.746, 95% CI: −1.019, −0.472), FT4/FT3 (β = −0.292, 95% CI: −0.401, −0.182), and TT4/TT3 (β = −0.009, 95% CI: −0.011, −0.007), while positively correlated with FT3 (β = 0.227, 95% CI: 0.178, 0.275), FT4/TT4 (β = 0.105, 95% CI: 0.067, 0.143), and FT3/TT3 (β = 0.001, 95% CI: 0.0004, 0.002). When the models were further adjusted for potential confounders, the associations with TT4 (β = −0.383, 95% CI: −0.695, −0.070) and TT4/TT3 (β = −0.003, 95% CI: −0.006, −0.0004) remained, but the association with FT3 (β = −0.129, 95% CI: −0.064, 0.039), FT4/FT3 (β = −0.111, 95% CI: −0.235, 0.012), FT4/TT4 (β= 0.021, 95% CI: −0.023, 0.065), and FT3/TT3 (β = 0.000, 95% CI: −0.001, 0.001) were no longer present.

**Table 2:** Association between dietary selenium intake and serum thyroid hormones in U.S. adults in NHANES 2007–2012.

	**Model 1**		**Model 2**	
	**β (95% CI)**	***p*-value**	**β (95% CI)**	***p*-value**
FT4	−0.146 (−0.469, 0.176)	0.366	−0.347 (−0.707, 0.013)	0.059
TT4	–**0.746 (**–**1.019**, –**0.472)**	**< 0.001**	–**0.383 (**–**0.695**, –**0.070)**	**0.017**
FT3	**0.227 (0.178, 0.275)**	**< 0.001**	−0.129 (−0.064, 0.039)	0.616
TT3	1.595 (−2.353, 5.543)	0.421	−1.807 (−6.464, 2.849)	0.439
TSH	−0.384 (−0.851, 0.084)	0.105	−0.232 (−0.679, 0.215)	0.303
FT4/FT3	–**0.292 (**–**0.401**, –**0.182)**	**< 0.001**	−0.111 (−0.235, 0.012)	0.077
TT4/TT3	–**0.009 (**–**0.011**, –**0.007)**	**< 0.001**	–**0.003 (**–**0.006**, –**0.0004)**	**0.024**
FT4/TT4	**0.105 (0.067, 0.143)**	**< 0.001**	0.021 (−0.023, 0.065)	0.348
FT3/TT3	**0.001 (0.0004, 0.002)**	**0.005**	0.000 (−0.001, 0.001)	0.707

FT4, free thyroxine; TT4, total thyroxine; FT3, free triiodothyronine; TT3, total triiodothyronine; TSH, thyroid-stimulating hormone. Model 1 (unadjusted) did not include any covariates. Model 2 was adjusted for sex, age, race, education level, household income, marital status, BMI, serum cotinine level, drinking, urine iodine concentration, and fasting time. The bold values indicate statistically significant differences.

Subgroup analyses by sex (Table 3) showed that the negative association between LogSe and TT4 (β = −0.007, 95% CI: −0.013, −0.001) and TT4/TT3 (β = −0.664, 95% CI: −1.182, −0.146) tended to be stronger in male adults compared to female adults. In addition, there was also a positive correlation between LogSe and FT4/TT4 (β = 0.031, 95% CI: 0.004, 0.059) in male adults.

**Table 3:** Association between dietary selenium intake and thyroid hormones in U.S. adults after subgroup analysis by sex.

**Outcomes**	**Male**		**Female**	
	**β (95% CI)**	***p*-value**	**β (95% CI)**	***p*-value**
FT4	−0.002 (−0.006, 0.002)	0.346	−0.005 (−0.011, 0.001)	0.106
TT4	–**0.007 (**–**0.013**, –**0.001)**	**0.032**	−0.003 (−0.008, 0.002)	0.242
FT3	−0.000 (−0. 021, 0.020)	0.975	−0.006 (−0.022, 0.011)	0.481
TT3	−0.000 (−0.000, 0.000)	0.756	−0.000 (−0.001, 0.000)	0.472
TSH	−0.002 (−0.007, 0.003)	0.404	−0.002 (−0.007, 0.003)	0.375
FT4/FT3	−0.007 (−0.018, 0.004)	0.205	−0.009 (−0.026, 0.007)	0.262
TT4/TT3	–**0.664 (**–**1.182**, –**0.146)**	**0.013**	−0.091 (−0.627, 0.444)	0.734
FT4/TT4	**0.031 (0.004, 0.059)**	**0.027**	−0.015 (−0.052, 0.022)	0.409
FT3/TT3	0.196 (−1.513, 1.904)	0.819	0.060 (−1.331, 1.452)	0.931

FT4, free thyroxine; TT4, total thyroxine; FT3, free triiodothyronine; TT3, total triiodothyronine; TSH, thyroid-stimulating hormone. Model was adjusted for sex, age, race, education level, household income, marital status, BMI, serum cotinine level, drinking, urine iodine concentration, and fasting time. The bold values indicate statistically significant differences.

Moreover, subgroup analysis by iodine status (Table 4) showed that LogSe was negatively associated with TT4 (β = −0.006, 95% CI: −0.011, −0.002), FT4/FT3 (β = −0.011, 95% CI: −0.023, −0.00002), and TT4/TT3 (β = −0.456, 95% CI: −0.886, −0.026) in iodine sufficiency but not in iodine deficiency adults.

**Table 4:** Association between dietary selenium intake and thyroid hormones in U.S. adults after subgroup analysis by urine iodine concentration.

**Outcomes**	**>100 ug/L**		≤ **100 ug/L**	
	**β (95% CI)**	***p*-value**	**β (95% CI)**	***p*-value**
FT4	−0.004 (−0.008, 0.000)	0.052	−0.002 (−0.008, 0.004)	0.436
TT4	–**0.006 (**–**0.011**, –**0.002)**	**0.009**	−0.004 (−0.012, 0.004)	0.319
FT3	0.000 (−0.009, 0.009)	0.963	−0.010 (−0.041, 0.020)	0.495
TT3	−0.000 (−0.001, 0.000)	0.302	0.000 (−0.000, 0.000)	0.909
TSH	−0.001 (−0.004, 0.003)	0.698	−0.004 (−0.010, 0.002)	0.217
FT4/FT3	–**0.011 (**–**0.023**, –**0.00002)**	**0.050**	−0.005 (−0.023, 0.013)	0.606
TT4/TT3	–**0.456 (**–**0.886**, –**0.026)**	**0.038**	−0.434 (−0.962, 0.093)	0.104
FT4/TT4	0.012 (−0.015, 0.039)	0.382	0.013 (−0.030, 0.056)	0.548
FT3/TT3	0.496 (−0.657, 1.649)	0.392	−0.541 (−3.326, 2.243)	0.698

FT4, free thyroxine; TT4, total thyroxine; FT3, free triiodothyronine; TT3, total triiodothyronine; TSH, thyroid-stimulating hormone. Model was adjusted for sex, age, race, education level, household income, marital status, BMI, serum cotinine level, drinking, urine iodine concentration, and fasting time. The bold values indicate statistically significant differences.

**Figure 2:**
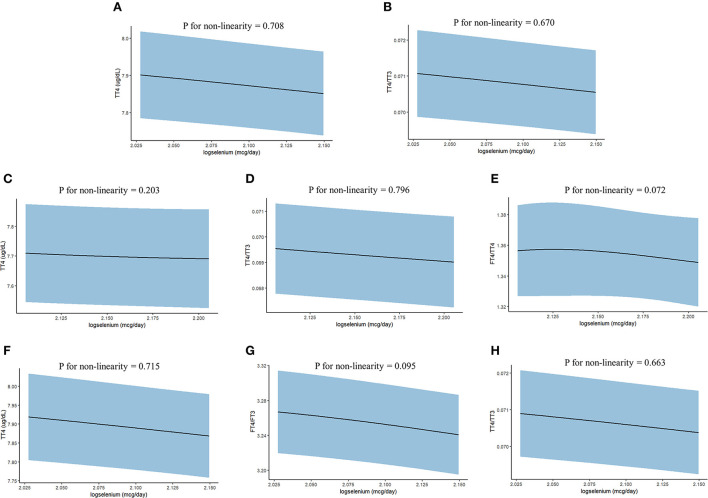
The dose-response relationship between dietary selenium intake and thyroid hormones in the whole and subgroup adults from NHANES 2007–2012. **(A)** TT4 of whole adults; **(B)** TT4/TT3 of whole adults; **(C)** TT4 of male adults; **(D)** TT4/TT3 of male adults; **(E)** FT4/TT4 of male adults; **(F)** TT4 of iodine-sufficient adults; **(G)** FT4/FT3 of iodine-sufficient adults; and **(H)** TT4/TT3 of iodine-sufficient adults. Point estimates (solid line) and 95% confidence intervals (blue area) were estimated by restricted cubic splines analysis with knots placed at the 5th, 25th, 50th, 75th, and 95th percentile. Models were adjusted for sex, age, race, education level, household income, marital status, BMI, serum cotinine level, drinking, urine iodine concentration, and fasting time.

In addition, we further used the restricted cubic splines to estimate the dose-response relationship between LogSe and thyroid hormones (Figure 2). Overall, there was not any departure from linearity in TT4 (*P for non-linearity* = 0.708) and TT4/TT3 (*P for non-linearity* = 0.670) of whole adults, TT4 (*P for non-linearity* = 0.203), TT4/TT3 (*P for non-linearity* = 0.796), and FT4/TT4 (*P for non-linearity* = 0.072) of male adults, and TT4 (*P for non-linearity* = 0.715), FT4/FT3 (*P for non-linearity* = 0.095), and TT4/TT3 (*P for non-linearity* =0.663) of iodine-sufficient adults.

## Discussion

Based on a nationally representative survey of non-institutionalized US adults, we found inverse correlations between LogSe and TT4 and TT4/TT3 in U.S. adults. LogSe was also negatively associated with FT4/FT3 and positively associated with FT3, FT4/TT4, and FT3/TT3 in U.S. adults. However, these correlations were no longer significant when the model was further adjusted for potential confounders. When the subgroup analysis was performed according to sex, we found that LogSe was negatively correlated with TT4 and TT4/TT3 while positively correlated with FT4/TT4 within male adults. When subgroup analysis was carried out according to urine iodine concentration, we found that LogSe was negatively associated with TT4, FT4/FT3, and TT4/TT3 within iodine sufficiency adults.

Previous research showed that low selenium status was associated with an increased risk of thyroid disease (32, 33). However, the association between selenium and thyroid hormones remains unknown. Contempré et al. found that selenium supplementation caused a decrease in serum T4 concentrations without a concomitant increase in serum TSH in healthy children (34). Consistent with the above findings, our results showed that LogSe was negatively correlated with serum TT4 but not with TSH in U.S. adults. The biological mechanism of the negative correlation between dietary selenium intake and TT4 has not been fully clarified. That may be because dietary selenium intake could increase type I deiodinase activity, which eventually reduces the concentration of T4 in the serum (35). Meanwhile, we also identified that LogSe negatively correlated with TT4/TT3, which further reflected that dietary selenium intake could contribute to the metabolism of peripheral T4. To fully understand the potential mechanism, further experiments *in vivo* and *in vitro* are needed in future studies.

Furthermore, the present study also found sex differences in the relationship between dietary selenium intake and thyroid hormone levels. For male adults, LogSe was negatively associated with TT4 and TT4/TT3 while positively correlated with FT4/TT4. Previous studies have demonstrated that estrogens could increase iodine uptake, thyroperoxidase activity, thyroglobulin expression, and modulate TSH levels (36–38). Moreover, estrogens also influence thyroid gland redox status by regulating nicotinamide adenine dinucleotide phosphate oxidase 4 (NOX4) and dual oxidase 2 (DUOX2) activity and expression (37). Selenium might have a much weaker effect on thyroid hormones than estrogens, suggesting that the effect of dietary selenium intake on thyroid hormones may be more significant in male adults. However, the sex differences between dietary selenium intake and thyroid hormone remain elusive, and further studies are needed to explore possible mechanisms.

Iodine is also an essential micronutrient for the thyroid gland to synthesize thyroid hormones (39). Previous studies have identified that both iodine deficiency and iodine excess may lead to thyroid dysfunction (40, 41). Thus, we carried out subgroup analyses stratified by urine iodine concentration. The results showed that LogSe was negatively associated with TT4, FT4/FT3, and TT4/TT3 in iodine sufficiency adults. Nevertheless, these connections were not observed in iodine-deficient adults. The major reason for explaining this result is that iodine deficiency results in reduced circulating TT3 and TT4 and increased TSH, which weakens the effect of dietary selenium intake on thyroid hormones (42).

Selenium is an essential nutrient element, which is rich in organ meats and seafood, followed by grain, cereals, and dairy products (43). However, it is worth noting that although selenium possesses various biological actions, such as anti-inflammatory and anti-oxidant properties, excessive selenium intake may also lead to harmful consequences. Multiple reports showed that high exposure to selenium was associated with increased risks of type 2 diabetes and non-alcoholic fatty liver disease (44, 45). Our results showed that LogSe was negatively correlated with serum TT4. Meanwhile, both thyroid hormone deficiency and excess may lead to a number of deleterious consequences including hyperthyroidism, hypothyroidism, thyroid inflammation, thyroid nodules, and thyroid cancer (46, 47). Furthermore, a diet intervention study showed that the high selenium diet could induce a subclinical hypothyroid response, while the low selenium diet could cause a subclinical hyperthyroid response (48). Therefore, to avoid excess risk, it is recommended that selenium-rich foods should be consumed carefully, considering individual dietary requirements.

The strength of the present study was that this was the first large population-based study to date to reveal the relationship between dietary selenium intake and thyroid hormones in U.S. adults to the best of our knowledge, and our findings might be a complement to the literature regarding the association between selenium and thyroid health. However, the study also had some limitations. First, as with any cross-sectional study, we cannot ascertain causality between dietary selenium and thyroid hormone. Second, dietary data were collected using two days of 24-h dietary recall survey, which might cause an underestimation or overestimation of diet selenium consumption. Third, the data on dietary selenium supplementation was not assessed due to substantial missing data. Lastly, we excluded 4,973 participants due to missing values for covariates, complete information about dietary selenium intake, or were under the minimum criteria on dietary recall status, which might decrease the generalizability of our results.

In conclusion, this study demonstrated that the increased dietary selenium intake was negatively correlated with TT4 and TT4/TT3 in U.S. adults. Furthermore, the association between dietary selenium intake and thyroid hormones was more pronounced in males and iodine sufficiency adults. However, further large-scale prospective studies are needed to confirm these findings in different populations.

## Data availability statement

The original contributions presented in the study are included in the article/supplementary material, further inquiries can be directed to the corresponding authors.

## Author contributions

FL and JN contributed to the conception and design of this study. FL, KW, and M-GD performed the statistical analysis. FL, QF, XL, and YY wrote the manuscript. All authors contributed to manuscript revision, read, and approved the submitted version.

## Conflict of interest

The authors declare that the research was conducted in the absence of any commercial or financial relationships that could be construed as a potential conflict of interest.

## Publisher's note

All claims expressed in this article are solely those of the authors and do not necessarily represent those of their affiliated organizations, or those of the publisher, the editors and the reviewers. Any product that may be evaluated in this article, or claim that may be made by its manufacturer, is not guaranteed or endorsed by the publisher.
